# Serum Osteoprotegerin Is an Independent Marker of Metabolic Complications in Non-DialysisDependent Chronic Kidney Disease Patients

**DOI:** 10.3390/nu13103609

**Published:** 2021-10-15

**Authors:** Aleksandra Rymarz, Katarzyna Romejko, Anna Matyjek, Zbigniew Bartoszewicz, Stanisław Niemczyk

**Affiliations:** 1 Department of Internal Diseases, Nephrology and Dialysis, Military Institute of Medicine, 128 Szaserów Street, 04-141 Warsaw, Poland; katarzyna.romejko@op.pl (K.R.); amatyjek@wim.mil.pl (A.M.); sniemczyk@wim.mil.pl (S.N.); 2 Department of Internal Diseases and Endocrinology, Medical University of Warsaw, 1a Banacha St., 02-097 Warsaw, Poland; zbigniew.bartoszewicz@wum.edu.pl

**Keywords:** chronic kidney disease, kidney, inflammation, protein energy-wasting, metabolic complications, bioimpedance spectroscopy

## Abstract

Background: Osteoprotegerin (OPG) belongs to the tumour necrosis factor superfamily and is known to accelerate endothelial dysfunction and vascular calcification. OPG concentrations are elevated in patients with chronic kidney disease. The aim of this study was to investigate the association between OPG levels and frequent complications of chronic kidney disease (CKD) such as anaemia, protein energy wasting (PEW), inflammation, overhydration, hyperglycaemia and hypertension. Methods: One hundred non-dialysis-dependent men with CKD stage 3–5 were included in the study. Bioimpedance spectroscopy (BIS) was used to measure overhydration, fat amount and lean body mass. We also measured the serum concentrations of haemoglobin, albumin, total cholesterol, C-reactive protein (CRP), fibrinogen and glycated haemoglobin (HgbA1c), as well as blood pressure. Results: We observed a significant, positive correlation between OPG and age, serum creatinine, CRP, fibrinogen, HgbA1c concentrations, systolic blood pressure and overhydration. Negative correlations were observed between OPG and glomerular filtration rate (eGFR), serum albumin concentrations and serum haemoglobin level. Logistic regression models revealed that OPG is an independent marker of metabolic complications such as anaemia, PEW, inflammation and poor renal prognosis (including overhydration, uncontrolled diabetes and hypertension) in the studied population. Conclusion: Our results suggest that OPG can be an independent marker of PEW, inflammation and vascular metabolic disturbances in patients with chronic kidney disease.

## 1. Introduction

Osteoprotegerin (OPG) is a molecule belonging to the tumour necrosis factor (TNF) superfamily. It is a decoy receptor for TNF-related apoptosis-inducing ligand (TRAIL). The TNF superfamily regulates immune responses, homeostasis as well as haematopoiesis [1]. Initially, OPG was identified as a bone resorption inhibitor that blocks the binding of RANK (receptor activator of nuclear factor kappa-β) to its ligand RANKL [2]. Further studies revealed that it has an additional impact on the cardiovascular and immune systems [3]. The OPG/RANKL/RANK system is involved in pathological angiogenesis, inflammatory states and cell survival. OPG is a glycoprotein released by vascular smooth muscle cells, endothelial cells, osteoblasts and immune cells. There is a signalling pathway between endothelial cells and osteoblasts during osteogenesis creating a connection between angiogenesis and osteogenesis [4]. The OPG/RANKL/RANK/TRAIL system, via receptors located on the cell surface, sends intracellular signals and modifies gene expression. As a result, monocytes, neutrophils and endothelial cells are recruited through cytokine production and receptor activation [5]. Serum OPG levels are associated with endothelial dysfunction and mediate vascular calcification [6]. Elevated OPG levels were observed in patients with atherosclerosis, heart failure, metabolic syndrome and diabetes [7,8,9].

The role of OPG in kidney pathologies is not well understood. OPG is expressed in kidney samples, cultured tubular cells and urinary exosome-like vesicles. It has been hypothesized that it can play a role in matrix deposition, inflammation and apoptosis [10]. Furthermore, the role of OPG in cardiovascular pathology and vascular calcification, which are common complications of chronic kidney disease, makes OPG an interesting marker also in CKD. When compared to healthy individuals, non-dialysis-dependent CKD patients, haemodialysis and peritoneal dialysis patients and renal transplant recipients present elevated levels of OPG [11,12,13]. As OPG is involved in vascular calcification, it is also associated with cardiovascular mortality in CKD patients [14]. Decreased levels of OPG are observed in nephrotic syndrome, which is probably caused by the loss with urine. Glucocorticosteroid treatment increases RANKL and reduces OPG expression, which also decreases the OPG levels in patients with nephrotic syndrome [15].

The purpose of our study was to investigate the association between OPG levels and the main complications of chronic kidney disease, including anaemia, protein energy wasting, inflammation and poor prognosis factors of CKD progression such as overhydration, hyperglycaemia and hypertension.

## 2. Methods

### 2.1. Design

A prospective, observational study in male patients with chronic kidney disease non-treated with dialysis was performed. The inclusion criterion was eGFR lower than 60 mL/min/1.73 m^2^.

### 2.2. Patients

Participants were recruited among patients visiting the Nephrological Outpatient Clinic for a routine control during the period between November 2018 and February 2020. If they fulfilled the inclusion criteria, a new visit was arranged for participation in the study. The participants were asked to avoid saunas, physical exertion and alcohol consumption the day before the examination. The visit took place after overnight fasting.

One hundred men with chronic kidney disease and eGFR lower than 60 mL/min/1.73 m^2^ were included in the study. The exclusion criteria were: renal replacement therapy or its requirement within the following 3 months, clinical signs of infection and presence of metal parts in the body. The study protocol was accepted by the local ethics committee (Bioethics Committee in Military Institute of Medicine, IRB acceptance number 120/WIM/2018 obtained 22 August 2018). All participants signed an informed consent.

Body composition including overhydration (OH), fat amount and lean body mass were measured by bioimpedance spectroscopy with the use of a Body Composition Monitor (Fresenius Medical Care). While being measured, patients stayed in a supine position after a 5 min rest. Electrodes were placed in a tetrapolar configuration (on one hand and one foot).

Blood samples for standard measurements were collected after an overnight fast and were transported immediately to the local Department of Laboratory Diagnostics. Concentrations of high-sensitivity C-reactive protein were determined by a nephelometry assay (BNII Siemens) with a cut-off point of 0.8 mg/dL. Serum creatinine concentrations were measured using the Jaffe method (Gen.2; Roche Diagnostics GmbH, Risch-Rotkreuz, Switzerland), and serum albumin levels using a BCP Albumin Assay Kit (Roche Diagnostics GmbH, Risch-Rotkreuz, Switzerland). Samples for measuring osteoprotegerin (OPG) levels were kept frozen at −80 °C. OPG levels were assessed using the Luminex MAGPIX platform.

GFR was calculated according to the short Modification of Diet in Renal Disease (MDRD) formula.

GFR in mL/min per 1.73 m^2^ = 175 × SerumCr^−1.154^ × age^−0.203^ × 1.212 (if patient is black) × 0.742 (if female)

### 2.3. Defining the Complications of Chronic Kidney Disease

Complications of chronic kidney disease were defined as follows:

- Anaemia, when serum haemoglobin level was lower than 12 g/dL [16]
- Protein energy wasting (PEW), when one of the following occurred: serum albumin level was lower than 3.8 g/dL, total cholesterol level was lower than 100 mg/dL, BMI was lower than 23 kg/m^2^ or Fat was lower than 10% [17]
- Inflammatory state, when CRP >0.8 mg/dL or Fbg >400 mg/dL [17]
- Poor prognostic factors of CKD progression, when HbA1c >8% or SBP >140 mmHg or presence of overhydration, i.e., (OH) >4 L [18]

### 2.4. Statistical Analysis

The characteristics of the study population are presented using medians with interquartile ranges (for continuous data with distribution other than normal, tested with the Shapiro–Wilk test). The correlation between OPG and clinical and laboratory parameters was assessed using the Spearman rank correlation coefficient. Logistic regression was used to investigate the significance of OPG levels as a marker of metabolic complications of CKD. Given the known relationship between OPG, age and GFR, we compared the value of logistic models, with and without OPG, for each metabolic complication (anaemia, PEW, inflammation, uncontrolled factors of CKD progression). Suitability of fit of each model was assessed using the area under the curve (AUC) with standard error (SE) and presented using the Receiver Operating Characteristic (ROC) curve. The models were compared using Hanley’s algorithm [19]. The analysis was performed with Statistica 13.1 with *p*-values < 0.05 considered statistically significant.

## 3. Results

The study population consisted of 100 male patients with chronic kidney disease and eGFR lower than 60 mL/min/1.73 m^2^, non-treated with dialysis. The median age of the studied population was 66 years (59–72). Clinical data are presented in Table 1.

We observed significant, positive correlations between OPG and age, serum creatinine concentration, CRP, fibrinogen, HgBA1C, systolic blood pressure and overhydration. Significant, negative correlations were observed between OPG and eGFR, serum albumin concentration as well as serum haemoglobin level. Table 2.

Logistic regression models, adjusted for age and GFR, were created to evaluate the influence of the OPG level as an independent marker of complications in chronic kidney disease patients, such as anaemia, PEW, an inflammatory state, poor prognostic factors of CKD progression.

### 3.1. Anaemia

The OPG level was a significant, independent marker of anaemia in the studied population (*p* < 0.001).

**Figure 1:**
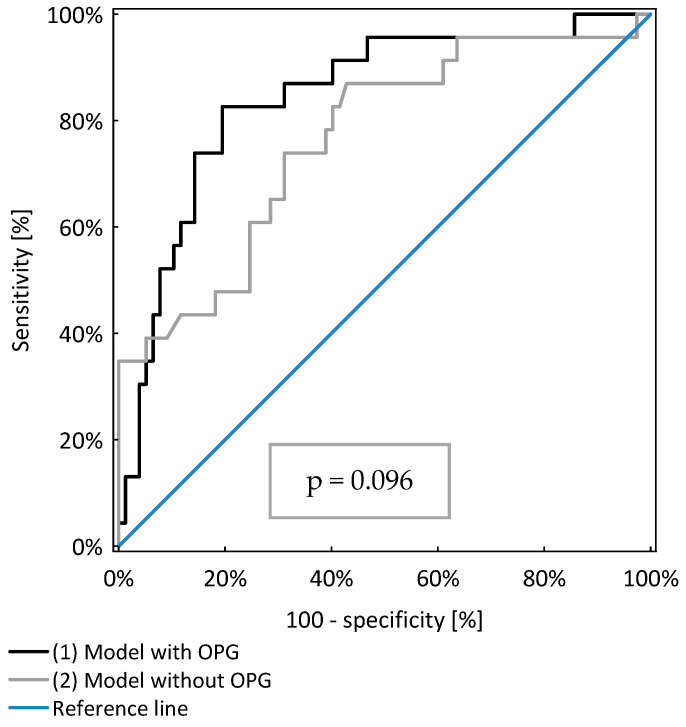
Comparison of two models of logistic regression analysis identifying CKD patients with anaemia.

The model including OPG, age and GFR (AUC=0.84 ± 0.05) identified CKD patients with anaemia better than the model including only age and GFR (AUC=0.76 ± 0.06), as shown in Table 3. However, the difference was not of statistical significance (*p* = 0.096), as shown in Figure 1.

### 3.2. Protein Energy Wasting (PEW)

**Figure 2:**
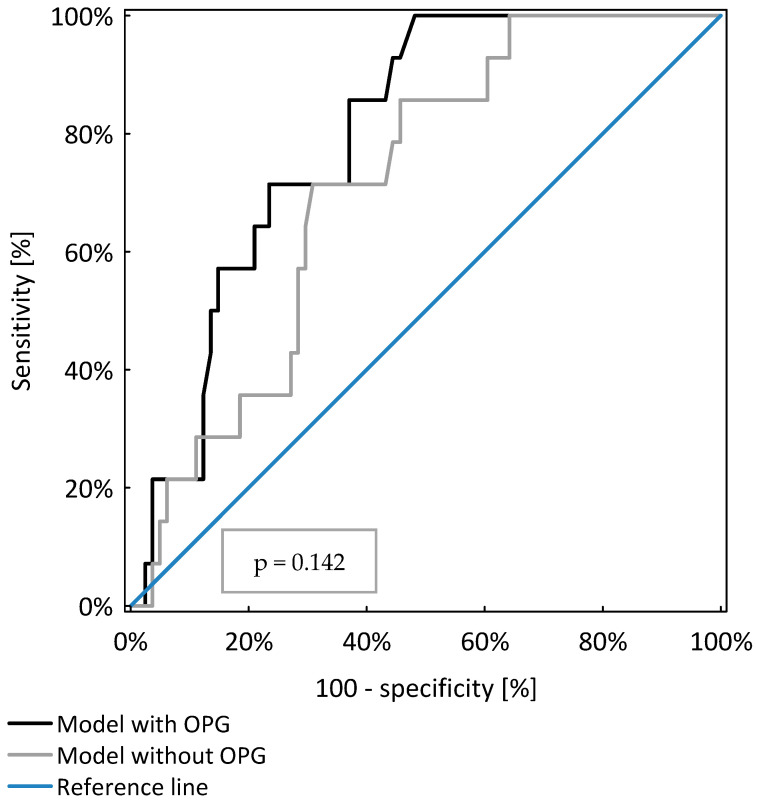
Comparison of two models of logistic regression analysis identifying CKD patients with protein energy wasting.

OPG was a significant, independent risk factor for PEW occurrence in the study population (*p* < 0.001). However, the model including OPG, age and GFR was not significantly better (AUC = 0.79 ± 0.05) than those considering only age and GFR (AUC = 0.71 ± 0.06, *p* = 0.142), as shown in Table 4 and Figure 2.

### 3.3. Inflammatory State

**Figure 3:**
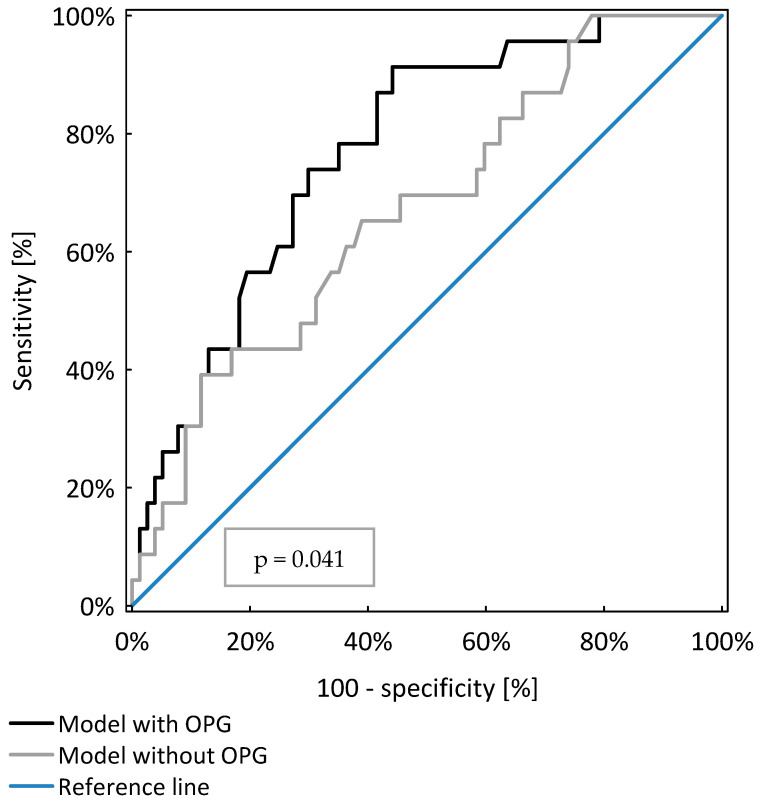
Comparison of two models of logistic regression analysis identifying CKD patients with protein energy wasting.

OPG was a significant, independent risk factor for subclinical inflammation in the study population (*p* < 0.001). The model including OPG, age and GFR was significantly better (AUC = 0.77 ± 0.05) than the model including only age and GFR (AUC = 0.67 ± 0.06; *p* = 0.041), as shown in Table 5 and Figure 3.

### 3.4. Poor Prognostic Factors (Overhydration, Hyperglycaemia and Hypertension)

**Figure 4:**
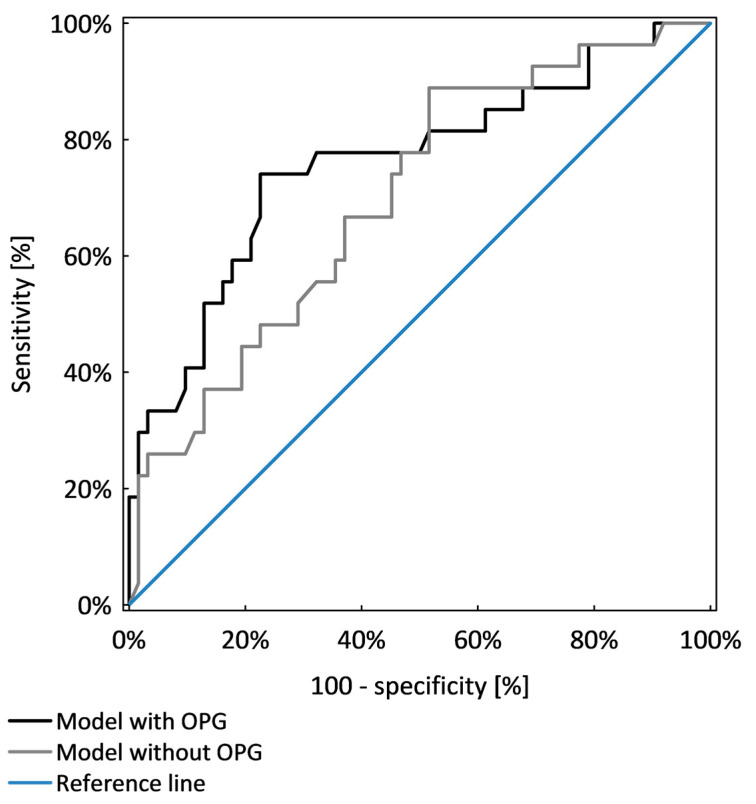
Comparison of two models of logistic regression analysis identifying CKD patients with poor prognostic factors.

Overhydration, hyperglycaemia and hypertension were chosen as poor prognostic factors of chronic kidney disease progression and were analysed together in logistic regression analysis. OPG was an independent marker that identified patients with poor prognostic factors (*p* < 0.001). The model including OPG, age and GFR was significantly better (AUC = 0.77 ± 0.05) than the model which included only age and GFR (AUC = 0.67 ± 0.06; *p* = 0.041), as shown in Table 6 and Figure 4.

## 4. Discussion

The role of OPG in chronic kidney disease patients is insufficiently comprehended. We know that the OPG level increases together with a decrease in the glomerular filtration rate, as proved in many studies [20,21]. The results of our study also confirm these observations. In a population of non-dialysis-dependent men with stage 3–5 chronic kidney disease, a significant, inverse correlation between OPG levels and eGFR was noted (R = −0.36; *p* < 0.001).

In our study, we also observed significant correlations between OPG and parameters which indicate CKD complications. The OPG level was correlated with the level of haemoglobins and acted as a marker of anaemia occurrence in the studied population. The logistic regression model including OPG was superior to that including only age and GFR in identifying patients with anaemia. However, the difference was not of statistical significance. OPG, as a member of the TNF superfamily, can influence haematopoiesis. Some researchers have observed that TNF superfamily members can regulate the growth of hematopoietic stem cells and progenitor cells [1,22].

Aside from anaemia, protein energy wasting (PEW) is also a complication of chronic kidney disease. The components of PEW are low serum albumin, cholesterol levels as well as low BMI and fat amount [17]. In our study, the OPG level was a marker of PEW in non-dialysis-dependent CKD men. PEW aetiology in CKD patients is associated with many factors such as hormonal disorders, insulin resistance and a subclinical inflammatory state. In our study, the OPG levels also correlated with the CRP and fibrinogen levels. OPG was also a significant marker of instances of inflammation. The logistic regression model that included OPG proved significantly better in in the identification of subclinical inflammation than the model which only included age and GFR. This finding is unsurprising, because OPG is known for its pro-inflammatory action. OPG has been described as a marker of endothelial damage associated with the inflammatory process, and in vitro studies have shown that OPG is involved in inflammatory cell chemotaxis [23].

Most studies concerning OPG in CKD patients concentrate on the association between OPG and vascular calcification. OPG levels correlate with vascular calcification in non-dialysis-dependent CKD patients as well as in patients treated with renal replacement therapy [24,25]. In animal studies, mice without OPG developed severe vascular calcification [26]. Therefore, it is hypothesized that OPG has a protective role in vascular injury, and the elevation of OPG levels inhibits vascular calcification. However, vascular calcification can also occur in the medial as well as in the intimal layer. In atherosclerosis, intima is thickened, inflamed and calcified, forming plaques with diffuse localisation along the vessel walls, whereas medial calcification occurs along the elastic lamina, leading to stiffness of the artery wall. Studies in animal models, including Apolipoprotein E-deficient mice, suggest that OPG plays a protective, anti-calcification role in both the medial and the intimal (atherosclerotic) calcification process [27].

The OPG levels increase rapidly in the early stages of vascular calcification and subsequently remain almost invariant [21]. Therefore, OPG appears to be a marker of the onset of atherosclerosis. However, the development of cardiovascular disease (CVD) begins with endothelial dysfunction. The earliest manifestation of CVD is observed in the area of microcirculation, and in CKD patients, this area is affected by the loss of homeostasis, even in cases of non-severe renal impairment [28]. OPG, as a factor released by endothelial cells, can be a marker of endothelial dysfunction and may probably be involved in a complicated chain of pathophysiological connections between chronic kidney disease and CVD.

In our study, we analysed factors of poor prognosis of CKD progression which are associated with vascular damage, such as overhydration, hypertension and glycaemic disturbances expressed by HgbA1c. Positive, significant correlations were observed between OPG levels and all three parameters. In other studies, OPG levels were associated with glycaemic status, and higher OPG levels were observed in patients with poor glycaemic control [29]. In our study, OPG was identified independently, associated with the presence of poor prognostic factors (overhydration, hypertension and glycaemic disturbances) irrespective of age and eGFR.

The limitation of our study is its relatively small sample size. An increase in the number of participants could enable a division of the study population into various subgroups according to the different stages of chronic kidney disease. Complications of CKD are more severe in more advanced stages of CKD, and OPG influence could also be more significant.

## 5. Conclusions

CKD is in itself a cardiovascular risk factor, and cardiovascular mortality in CKD patients is high. CVD mortality in CKD patients is even greater in the presence of diabetes and hypertension [30]. Early detection of such complications and the identification of patients at risk of developing them is vital. Therefore, new markers that can predict metabolic disorders and have an unfavourable effect on vascular damage are necessary. OPG seems to be a possible marker associated with PEW, inflammation and vascular metabolic disturbances which is worth further study.

## Figures and Tables

**Table 1:** Clinical characteristics of the study population.

	Median	IQR
Age [years]	66	59–72
Serum Creatinine concentration [mg/dL]	1.9	1.5–2.7
eGFR [mL/min/1.73 m^2^]	38.4	25.4–48.9
Hgb [g/dL]	13.4	12.1–14.6
Serum albumin concentration [g/dL]	4.4	4.1–4.6
Total cholesterol [mg/dL]	165	143–207
BMI [kg/m^2^]	28.6	25.4–33.3
Fat [%]	29.8	24.1–36.7
CRP [mg/dL]	0.2	0.1–0.4
Fibrinogen [mg/dL]	339	268–419
HgbA1c [%]	5.8	5.3–6.4
Systolic blood pressure [mmHg]	130	125–140
Overhydration OH [L]	−0.1	−0.9–1.2
Osteoprotegerin [pg/mL]	425.6	300–556.3

BMI, body mass index; CRP, C-reactive protein; eGFR, estimated glomerular filtration rate; Hgb, haemoglobin; HgbA1c, haemoglobin A1c.

**Table 2:** Correlations between OPG and the studied parameters.

OPG	R	*p*
Age	**0.44**	**<0.001**
Serum Creatinine concentration	**0.34**	**<0.001**
eGFR mL/min/1.73 m^2^	**−0.36**	**<0.001**
Hgb	**−0.52**	**<0.001**
Serum albumin concentration	**−0.37**	**<0.001**
Total cholesterol	**−0.21**	**0.033**
BMI	0.01	0.869
Fat	**0.21**	**0.045**
CRP	**0.33**	**<0.001**
Fibrinogen	**0.39**	**0.002**
HgbA1c	**0.48**	**<0.001**
Systolic blood pressure	**0.30**	**0.002**
Overhydration OH [L]	**0.34**	**0.001**

BMI, body mass index; CRP, C-reactive protein; eGFR, estimated glomerular filtration rate; Hgb, haemoglobin; HgbA1c, haemoglobin A1c, R, Spearman rank correlation coefficient. The significant *p* and R values are bolded.

**Table 3:** Markers associated with anaemia in chronic kidney disease patients based on a logistic regression model.

Parameter	B ± SE	OR (95% CI)	*p*-Value of Variable	H–L *p*-Value	AUC ± SE	*p*-Value of Model
Intercept	0.545 ± 1.755	-	0.756			
Age	−0.043 ± 0.032	0.958 (0.899–1.021)	0.184			
OPG	0.006 ± 0.002	1.006 (1.002–1.021)	0.004	0.749	0.84 ± 0.05	<0.001
eGFR	−0.052 ± 0.023	0.95 (0.909–1.021)	0.023			
Intercept	0.847 ± 1.641	-	0.606			
Age	0.008 ± 0.025	1.008 (0.959–1.059)	0.756	0.150	0.76 ± 0.06	<0.001
eGFR	−0.077 ± 0.021	0.926 (0.889–0.964)	<0.001			

**Table 4:** Markers associated with protein energy wasting in chronic kidney disease patients based on a logistic regression model.

Parameter	B ± SE	OR (95% CI)	*p*-Value of Variable	H–L *p*-Value	AUC ± SE	*p*-Value of Model
Intercept	−1.254 ± 2.082	-	0.547			
Age	−0.023 ± 0.035	0.977 (0.912–1.048)	0.522			
OPG	0.004 ± 0.002	1.004 (0.999–1.008)	0.051	0.544	0.79 ± 0.05	<0.001
eGFR	−0.029 ± 0.025	0.971 (0.925–1.019)	0.234			
Intercept	−0.676 ± 1.984	-	0.726			
Age	0.011 ± 0.030	1.011 (0.953–1.073)	0.724	0.466	0.71 ± 0.06	<0.001
eGFR	−0.052 ± 0.022	0.949 (0.910–0.991)	0.018			

eGFR, estimated glomerular filtration rate; OPG, osteoprotegerin.

**Table 5:** Markers associated with inflammation in chronic kidney disease patients based on a logistic regression model.

Parameter	B ± SE	OR (95% CI)	*p*-Value of Variable	H–L *p*-Value	AUC ± SE	*p*-Value of Model
Intercept	−4.302 ± 2.009	-	0.032			
Age	0.012 ± 0.031	1.012 (0.952–1.076)	0.693			
OPG	0.005 ± 0.002	1.005 (1.002–1.009)	0.005	0.703	0.77 ± 0.05	<0.001
GFR	−0.007 ± 0.021	0.993 (0.952–1.036)	0.748			
Intercept	−3.238 ± 1.798	-	0.072			
Age	0.050 ± 0.027	1.052 (0.997–1.109)	0.063	0.520	0.67 ± 0.06	<0.007
GFR	−0.036 ± 0.018	0.964 (0.931–0.999)	<0.045			

eGFR, estimated glomerular filtration rate; OPG, osteoprotegerin.

**Table 6:** Markers associated with poor prognostic factors in chronic kidney disease patients based on a logistic regression model.

Parameter	B ± SE	OR (95% CI)	*p*-Value of Variable	H–L *p*-Value	AUC ± SE	*p*-Value of Model
Intercept	−0.715 ± 1.677	-	0.670			
Age	−0.020 ± 0.030	0.980 (0.924–1.039)	0.499			
OPG	0.005 ± 0.001	1.005 (1.002–1.009)	0.006	0.625	0.77 ± 0.05	<0.001
GFR	−0.034 ± 0.20	0.966 (0.928–1.006)	0.093			
Intercept	−0.301 ± 1.578	-	0.849			
Age	0.022 ± 0.025	1.022 (0.974–1.073)	0.373	0.719	0.67 ± 0.06	0.007
GFR	−0.057 ± 0.019	0.944 (0.911–0.980)	0.002			

eGFR, estimated glomerular filtration rate; OPG, osteoprotegerin.

## Data Availability

All relevant data analyzed during the current trial are included in the article. Access to raw datasets may be provided upon reasonable request to the corresponding author following permission by the Ethics Committee and the Institute at which the study was conducted.
